# Harmonization issues in unit costing of service use for multi-country, multi-sectoral health economic evaluations: a scoping review

**DOI:** 10.1186/s13561-022-00390-y

**Published:** 2022-08-03

**Authors:** Claudia Fischer, Susanne Mayer, Nataša Perić, Judit Simon

**Affiliations:** 1 Department of Health Economics, Center for Public Health, Medical University of Vienna, Kinderspitalgasse 15/1, 1090 Vienna, Austria; 2 Department of Psychiatry, University of Oxford, Warneford Hospital, Oxford, OX3 7JX UK

**Keywords:** Valuation, Unit cost, Economic evaluation, Health and social care, Education, (criminal) justice, Societal perspective

## Abstract

**Background:**

Valuation is a critical part of the costing process in health economic evaluations. However, an overview of specific issues relevant to the European context on harmonizing methodological requirements for the valuation of costs to be used in health economic evaluation is lacking. We aimed to inform the development of an international, harmonized and multi-sectoral costing framework, as sought in the European PECUNIA (ProgrammE in Costing, resource use measurement and outcome valuation for Use in multi-sectoral National and International health economic evaluAtions) project.

**Methods:**

We conducted a scoping review (information extraction 2008–2021) to a) to demonstrate the degree of heterogeneity that currently exists in the literature regarding central terminology, b) to generate an overview of the most relevant areas for harmonization in multi-sectoral and multi-national costing processes for health economic evaluations, and c) to provide insights into country level variation regarding economic evaluation guidance. A complex search strategy was applied covering key publications on costing methods, glossaries, and international costing recommendations augmented by a targeted author and reference search as well as snowballing. Six European countries served as case studies to describe country-specific harmonization issues. Identified information was qualitatively synthesized and cross-checked using a newly developed, pilot-tested data extraction form.

**Results:**

Costing methods for services were found to be heterogeneous between sectors and country guidelines and may, in practice, be often driven by data availability and reimbursement systems in place. The lack of detailed guidance regarding specific costing methods, recommended data sources, double-counting of costs between sectors, adjustment of unit costs for inflation, transparent handling of overhead costs as well as the unavailability of standardized unit costing estimates in most countries were identified as main drivers of country specific differences in costing methods with a major impact on valuation and cost-effectiveness evidence.

**Conclusion:**

This review provides a basic summary of existing costing practices for evaluative purposes across sectors and countries and highlights several common methodological factors influencing divergence in cost valuation methods that would need to be systematically incorporated and addressed in future costing practices to achieve more comparable, harmonized health economic evaluation evidence.

**Supplementary Information:**

The online version contains supplementary material available at 10.1186/s13561-022-00390-y.

## Background

The increased health needs and demands as well as the scarcity of resources have resulted in a more prominent role of economic considerations in evidence-based healthcare decision-making [1]. Nevertheless, conducting economic evaluations (EEs) in the healthcare setting is a complex task. For instance, a prerequisite for the validity and usability of health economic evidence is sound costing methodology. Next to the identification, definition and measurement of relevant resource use for consideration in EEs [2], a critical part of the costing process is the valuation method. Valuation implies that the different resources used for the production of a unit of resource use (e.g. service) are multiplied with their value (e.g. price) and summed up to derive its cost, also referred to as unit cost. From an economic perspective, there is a consensus that the derived unit costs should ideally capture the benefit forgone when a resource is consumed, i.e. the opportunity cost [3], also referred to as the true ‘economic cost’ [4]. However, due to the influence of governmental regulations or stakeholder negotiations, the health and social care sectors are typically considered imperfect markets and e.g. hospital charges, physician fees or drug prices do not necessarily reflect opportunity costs [4].

While these unit costs and valuation sources have nonetheless been accepted for use in EEs in the healthcare sector [5], the application of different costing approaches and definitions still seem to be ambiguous [6]. The lack of harmonization of methodological requirements for cost valuation results in numerous practical challenges in relation to costing of health-related services, with many of these challenges not resolved. For example, no universal gold standard seems to exist up to date regarding the choice of overhead allocation method [2]. An important determinant for the appropriate source of valuation is the analytical (study) perspective from which the EE (economic evaluation) is conducted. The study perspective (e.g. a) provider, b) third-party payer, c) patient, d) society (i.e. the broadest perspective [7, 8]) determines which cost components (e.g. healthcare costs, patient and family out-of-pocket expenses, costs occurring in other sectors) are to be included in an EE [3]. At the same time, it also determines which sources are appropriate to value the service use [3]. For instance, charges may well reflect the costs from a payer’s perspective, while not necessarily capture the true economic cost of service provision from a societal perspective. Overall, several comparisons have shown that unit cost estimates in the healthcare sector are sensitive to the applied costing method (e.g. [9–11]).

The importance of harmonization of methodological requirements in relation to costing increases when healthcare interventions influence resource use outside the healthcare sector [12]. These so-called intersectoral costs and benefits [13] include, e.g. the valuation of spill over effects on employment and work productivity. A review shows that substantial differences in the items considered and methods used are resulting in major differences in lost productivity estimates [14]. This applies likewise to patient and family costs [15]. Although still a relatively new field of research, costs in the education and (criminal) justice sectors were found to be a vital cost component in EEs conducted from the societal perspective [16], especially in the field of mental health.

In light of the methodological heterogeneity, it often remains unclear if differences in (unit) costs observed between national and international studies are e.g. due to differences in applied costing methodology or differences attributable to the service [17]. From a global viewpoint, such differences could indeed alter the cut-off point between an efficient versus a non-efficient intervention in an EE, and eventually the decision on its reimbursement and implementation. To achieve more comparability and harmonization in methods across studies, countries and sectors, identification of the areas of controversy in cross-sectoral and cross-country cost valuations is a fundamental prerequisite. This insight is a vital first step, even if differences in data availability and formal requirements regarding the analytic perspective remain heterogeneous. Beyond this, methodological harmonization is also a relevant stepping-stone towards the increased transferability of EEs internationally.

The overarching objective of this current scoping review was to inform the development of an international, harmonized and multi-sectoral costing framework, as sought in the European PECUNIA (ProgrammE in Costing, resource use measurement and outcome valuation for Use in multi-sectoral National and International health economic evaluAtions) project [18]. This review covers multiple sectors including the health and social care, criminal justice and education sectors, as well as several selected countries as represented in the PECUNIA project. The specific objectives of the current scoping review were threefold:

- a) To establish a set of definitions of central economic terms and main concepts to determine the degree of heterogeneity that currently exists in the literature;
- b) To generate an overview of the most relevant potential areas for harmonization in multi-sectoral and multi-national costing processes for health EEs;
- c) To provide insights into country level variation regarding EE guidance, based on a case study of six European countries represented in the PECUNIA project.

## Methods

This scoping review was guided by the approach to map the main concepts, theories, evidence, knowledge gaps and their main sources as recommended by the Enhancing the QUAlity and Transparency Of health Research (EQUATOR) network [19]. The reporting checklist by the Preferred Reporting Items for Systematic reviews and Meta-Analyses extension for Scoping Reviews (PRISMA-ScR) was followed and can be found in the Additional file 1.

### Search strategy

With key grey literature and scientific peer-reviewed articles including multiple sectors (i.e. health and social care, criminal justice and education) and methodological aspects regarding costing not being always clearly identifiable as such, a comprehensive search strategy was required.

For the establishment of a set of definitions of central economic terms and main concepts to determine the degree of heterogeneity currently existing in the literature (research aim a)) we screened textbooks and online glossaries. In addition, we screened all sources identified in course of the literature search for research aim b) for definitions. The selection of the economic terms included in the glossary was based on the criteria ‘relevance’ and ‘likelihood for interpretation differences’, determined by the research team, who are experts in the field of health economics. This overview does not make any claim to comprehensiveness (i.e. providing a complete picture regarding all existing definitions and descriptions represented in the literature), but rather aims to highlight the potential variation and partial contradictions of existing definitions of costing approaches and their components. For the identification of general methodological issues regarding sector-specific valuation issues (research aim b) potentially causing amenable heterogeneity in service use costing, the following approach was adopted: Publications from previous EU projects linked to costing methods were screened (i.e. IMPACT HTA, HealthBASKET, IBenC [20–22]). Reference lists of these identified publications were screened for peer-reviewed publications and grey literature. An author and snowball search in Embase and PubMed were conducted to further identify relevant peer-reviewed methodological publications including books. In addition, relevant health economic and HTA websites were searched including the International Society for Pharmacoeconomics and Outcomes Research (ISPOR) [23], the international Health Economics Association (iHEA) [24], and the Unit Costs Article database collated by the Personal Social Services Research Unit (PSSRU) at the University of Kent.

For the identification and analysis of country-specific harmonization issues (research aim c)), six European countries represented in the European PECUNIA project (i.e. Austria, Germany, Hungary, Spain, the Netherlands and England), were selected as case studies for feasibility reasons. Since these countries represent different types of health care systems (i.e. tax-funded/social insurance-funded, different levels of costing guidance for EEs and use of such evidence, availability of standardized unit cost catalogues, geographic locations within Europe) the overview is nevertheless expected to provide a fairly complete picture. At the same time, broader international insights were considered where identified. As the development of costing methods and relevant recommendations is often part of the national Health Technology Assessment (HTA) agenda, national websites were considered as most promising to screen for country-specific guidance (i.e. national EE guidelines and unit cost(ing) manuals, costing guidelines and unit cost programmes).

The searches were augmented by additional literature (e.g. national grey literature) from the PECUNIA consortium and scientific advisory board (SAB).

### Study selection

The search was conducted throughout 2018 (the start of the PECUNIA project) and information extraction limited to publications from the past 10 years (i.e. 2008) to cover timely methods. Full-text publications of peer-reviewed articles and books as well as grey literature were included. In regards to EE guidelines, the latest available publications within the defined search window were considered. In addition, we conducted an update of the search until 2021 to include any major advancements after 2018 (i.e. updates of costing guidelines).

Textbooks, online glossaries, peer-reviewed publications and grey literature were selected if they included a relevant definition/description of central economic terms. Relevant health economic and HTA websites, peer-reviewed and grey literature were selected if they included main concepts or general methodological issues regarding sector-specific valuation issues, which are of relevance when conducting multi-country, multi-sectoral health EEs. National EE/HTA guidelines and manuals, available costing programmes/manuals and guidelines, and country-specific health economic and HTA websites were selected in case they included country-specific information and recommendations regarding valuation aspects for EEs.

Publications in German, Hungarian, Spanish, Dutch, and English were considered for inclusion.

### Data extraction

Identified publications were screened for their definitions/descriptions of central economic terms and main concepts. These were collected, compared and organized in a table format. General methodological aspects in respect to sector-specific valuation issues were extracted and grouped according to the specific issue it related to. For the identification and analysis of country-specific harmonization issues, data extraction was conducted using a newly developed and standardized, pilot-tested data extraction form created in MS Excel. Synthesised information on service costing methods recommendations covered: analytic study perspective (e.g. healthcare, societal), costs (to be included in an EE), list of national/regional unit cost lists available, source of valuation (cost data), the hierarchy of data sources stated for valuation, costing methodology (valuation of costs), measurement of capital costs, overhead costs and operating costs (e.g. capital costs), sources used to calculate reference prices for various healthcare services (e.g. public statistics), physical units (any common units used like hours of nursing; per visit), reporting of costs (any requirement to report costs and quantities/volumes separately), time horizon, discounting, adjustment for inflation/price indexation, specific considerations per field/area of application, periodicity of costing manual, recommended level of guideline adherence (e.g. mandatory).

In each stage of the applied methodology, at least two researchers were involved. The searches in the multiple information sources were executed by two researchers (CF, NP). Identified literature was screened against the above pre-defined inclusion criteria by two researchers (CF, NP). Potential disagreements regarding inclusion were resolved by discussion or if needed, by a third reviewer (SM, JS).

## Results

The initial search via previous EU projects (*N* = 35), relevant books (*N* = 2), and grey literature (*N* = 14) resulted in 51 hits. The search for EE guidelines, unit cost(ing) manuals/programmes/list of unit costs, health economic and HTA websites added 46 hits. This resulted in the inclusion of a total of 97 sources for information synthesis. The overall study selection process is illustrated in the PRISMA flow diagram (see Additional file 1).

### Heterogeneity of definitions of central economic terms and main concepts

Varying definitions and interpretations can be found in the international context, as the understanding and usage of a definition are highly dependent on the country context and the analysist’s professional background. Table 1 presents definitions and descriptions of central economic terms and main concepts. The aim of this glossary of terms is to give an overview of the potential variation and partial contradictions of existing definition of costing approaches and their components, which may be a jeopardizing factor for harmonization efforts.

**Table 1:** Glossary of identified definition(s) of central economic terms and main concepts used in the literature and definitions for costing

Term	Examples of definition/description(s) used in the literature
Average cost:	*-Total resource cost, including all support and overhead costs, divided by the total units of output *[25] *-Total cost divided by the number of units of output *[26]
Bottom-up costing:	-*For cost valuation, in the bottom-up approach, cost components are valued by identifying resource used directly employed for a patient* [27] *-The bottom-up approach assesses the amount of each resource that is used to produce an individual service and assigns costs accordingly to generate aggregate costs of a system* [28] *-Bottom-up approaches, such as activity-based costing, assess the amount of each resource that is used to produce an individual healthcare service and assigns costs accordingly to generate aggregate costs for a healthcare system* [29] -*To value cost items using the bottom-up approach, patient utilization data needs to be multiplied by unit prices, leading to cost estimates for individual patients *[3]
Capital cost:	- *The cost to purchase the major capital assets required by the programme (for example, equipment, buildings, and land) *[26, 30] -*Capital costs are one-time expenses typically incurred to set up a service *[3]
Fee:	-*A payment made to a professional or public organization for advice or services* [2] -*The amount charged for a resource or service* [31]
Fixed cost:	-*Fixed costs do not vary with the quantity of output in the short run (about 1 year) and vary with time, rather than quantity: e.g. rent, equipment lease payments, some wages and salaries *[3] *-Fixed cost is the one that remains stable regardless of the amount of production output and is actually the running cost of the department and the cost of equipment. Fixed cost is determined by staff salaries, capital and maintenance costs* [32]
(Bottom up/top down) gross costing:	-*In gross costing, cost components are defined at a highly aggregated level* [27] -*Bottom up gross costing values the cost component for each individual patient* [33] -*Top down gross costing values the cost component per average patient by separating out costs from comprehensive sources* [33]
Top-down costing:	-*In the top-down approach, cost components are valued by separating out the relevant costs from comprehensive sources* [27] -*The top-down approach relies on comprehensive sources, such as annual financial accounts, and divides aggregated costs by the total number of patients* [34] -*The step-down method, also known as the top-down method, calculates the unit cost of healthcare services by allocation of the total hospital cost* [35] -*Top-down methods work with aggregate expenditures, which reflect monetary flows at the service level rather than the value of resources used. This implicitly accepts prevailing prices or charges as the correct valuation of resource inputs* [5]
(Top down/bottom up) micro-costing:	-*For cost identification, in microcosting, all cost components are defined at the most detailed level* [27] *-A detailed list of each component of a patient’s care is created and costed separately for each facet of a patient’s hospitalization* [9] *Bottom-up micro-costing identifies all relevant cost components and values each cost component for all individual patients resulting in the most accurate cost estimates* [33] *Top down micro-costing identifies all relevant cost components, but values each component for average patients by separating out costs from comprehensive resources such as annual accounts* [33]
Opportunity costs:	-*Benefits forgone* [3] *- The cost of a unit of a resource is the benefit that would be derived from using it in its best alternative use.* [26] *- The opportunity cost of an intervention is what is foregone as a consequence of adopting a new intervention.* [36] - *The ‘value of the next-best alternative’ forgone […]* or *‘the value of what is given up’* [37]
Overhead/ indirect costs:	-*Overhead costs, which consist of employee benefits, administrative staff, and capital costs such as building and equipment operation and maintenance, cannot be directly attributed to patient care, nor are they as responsive to changes in patient volume as variable, direct costs* [38] -*Indirect cost components generally concern overheads (general expenses, administration and registration, energy, maintenance, insurance and the personnel costs of non-patient services…) and capital (depreciation of buildings and inventory and interest)* [33]
Unit cost:	-*The value of all resources (input) used to produce a service, divided by the level of activity (output) it generates* [39] -*Standard unit costs are defined as all costs related to the provision of a particular service* [40]
Variable cost:	-*Variable cost is designated by the activities necessary for each patient’s treatment and it includes the cost of medication, consumables and diagnostic tests* [32] - *Costs are often categorised into different types, such as […] fixed and variable costs (reflecting the initial payment for equipment and the additional cost per use of the consumables).* [36] - *Those costs which vary with the level of production and are proportional to quantities produced.* [41]

### General methodological issues regarding sector-specific valuation

The following paragraphs describe the identified general sector-specific methodological aspects and cross-sectoral costing challenges, including problems as well as potential solutions and recommendations, found in the screened literature. Table 2 provides an overview of these findings and highlights the multiple open questions regarding essential methodological aspects.

**Table 2:** Overview of identified challenges and potential solutions/recommendations by sector-specific or cross-sectoral methodological aspects

Health and social care sectors		
**Methodological aspect**	**Challenges described in the literature**	**Potential solutions described in the literature**
**Typical cost identification and valuation approaches:** top-down, bottom-up, micro-costing, gross-costing	Indistinctness and inconsistency regarding methods definitions as well as their application;	
	Practical feasibility hampers wide application of bottom-up micro-costing (time-consuming, unavailable information);	Consideration of bottom-up methodology at least for healthcare services with large component of overheads;
**Costing sources to develop unit costs**: reference unit costs, fees, charges, market prices	Lack of clarity regarding the difference of standard unit costs and market prices/charges and which costing perspective to apply;	
	Unit cost estimates are sensitive to the applied costing methods; size of impact of the different choices is unclear;	
	Ambiguity regarding the costing perspective to apply;	
	Country specific reference unit costs are the preferred proxy measure for the opportunity costs of health and social care services are, however, often unavailable	
**International comparison of unit costs:**	Internationally, there are large differences between salaries of professionals and diverse professions delivering the same service, potentially resulting in varying unit costs	Consideration of bottom-up methodology in multi-country studies with large differences between salaries of professionals and diverse professions delivering the same service;
**Costing methods:** total costs (fixed and variable), marginal costs	Ambiguity regarding cost types and components to consider;	Depending on the purpose, consideration of different time horizons and consequently cost components: Variable costs for services happening within existing infra structure despite requiring new investments on other levels; Marginal costs for services that can be offered by using existing equipment;
**Methods for valuation of overhead costs of services:** allocation of weighted service/hourly rate/inpatient day/marginal mark-up	Absence of universally accepted standard for the estimation of overhead costs;	Micro-costing is not feasible for the determination of overhead costs for hospitals/large institutions; Application of the ratio of overhead to direct expenses for similar departments;
**Education and criminal justice sectors**		
**Valuation of health-related service use in the education and (criminal) justice sectors**	Valuation methods of health-related service use in the education and (criminal) justice sector are less established. Their feasibility in different countries has yet to be determined;	Methodological choice should be based on the underlying data and their availability/ reliability;
	Feasibility to use opportunity cost method based on micro-costing is limited, as time consuming;	
	Reliability, transparency, unrestricted availability and transparent referencing are a prerequisite for the validity of utilizing market prices from governmental reports to calculate proxy unit prices, which is not always possible to determine	
**Cross-sectoral costing issues**		
**Perspective to adopt to consider all relevant costs/cross-sectoral costs**: societal perspective	Risk of double-counting due to lack of transparency in costing components;	

#### Methodological issues in costing for the health and social care sectors

Common health economic costing methods of services in the health and social care sectors are micro or gross costings for cost identification, and bottom-up or top-down approaches for valuation [22, 42–44]. Based on the available definitions of these approaches as described in different sources (e.g. [6, 45] however, it becomes apparent that these methods are not always clearly distinguishable or uniformly defined/applied [6, 18, 42]. In addition to the application of valuation approaches to newly developed unit costs, readily available costing sources may be used for the valuation of service use, including reference unit costs, fees, charges and market prices [22, 46]. According to recently published cross-European recommendations regarding the valuation of service use in EEs, the preferred proxy measure for the opportunity costs of healthcare and supportive care/social care services are country-specific reference unit costs, when available [40]. It remains unclear, however, in what way standard unit costs differ from market prices or charges, and e.g. what costing perspective (i.e. long-run, short-run) shall be incorporated in these estimates. At the same time, the chosen method is crucial for the resulting unit cost estimate [47]. Top-down and bottom-up approaches were previously found to yield different results when calculating the unit cost of cognitive behavioural therapy [47], although the size of the impact on the results was not specified. Also other comparisons confirmed the sensitivity of unit cost estimates in the healthcare sector regarding the applied costing methodology (e.g. [9, 11]).

Bottom-up micro-costing has not been widely used in assessing the costs of healthcare services [48]. Presumably, this is mainly due to its feasibility, as this methodology is time-consuming, especially when information systems are absent or inadequate [6]. Hospital costing studies indicate that a full bottom-up methodology should be considered for healthcare services with a large component of labour or overheads as expected for mental health services. In multi-country studies, international differences between salaries of comparable professionals may have significant effects on the unit costs. Moreover, in different countries diverse professions may deliver the same services resulting in further variation in the unit cost of specific services [47, 49–52].

It is generally agreed that depending on the specific service and its role in the EE, different costing methods are appropriate [3]. Besides, depending on the purpose of the costing exercise, different time horizons and hence cost components may be considered [3, 46]. For example [53], total costs (fixed plus variable costs) are relevant whenever a service requires considerable constructional changes (e.g. addition of a new operating theatre). Using variable costs is recommended for services that happen within existing infrastructure despite requiring new investments at other levels. Marginal costs are to be considered for services that can be offered by using existing equipment, while average costs capture total costs per unit of output [53].

Overhead costs may largely vary between different organisations, as do the use of services and the method of cost allocation [2]. Generally, there are different types of overhead costs such as capital versus non-capital to consider separately. They may be related exclusively to the management and administrative services, or to maintenance (e.g. catering, cleaning, gas, water) [2]. There are also several methods for performing overhead calculations which include the allocation of i) weighted service, ii) hourly rate, iii) inpatient day, or iv) marginal mark-up [54]. The weighted service method establishes the relative cost of the individual patient. Hourly rate yields a cost per treatment minute by employing service time of the primary treatment as a proxy for consumption. When using inpatient day allocation, all patients are assumed to have the same indirect costs per day irrespective of the actual resource use. In marginal mark-up allocation, indirect costs are distributed to direct costs by raising the direct costs with a mark-up percentage. [33].

Micro-costing is not feasible for the determination of overhead costs for hospitals and other large institutions [4]. Instead, it is suggested to apply the ratio of overhead to direct expenses for a similar subdivision [4]. Nevertheless, to date, there is no universally accepted standard for the estimation of overhead costs [2]. Potential double-counting of costs is another risk acknowledged in the health and social care sectors. For example, double counting may occur when financing costs are captured in the healthcare unit cost on the one hand but also separately considered as cost on the other hand [5, 55].

#### Methodological issues in costing for the education and criminal justice sectors

Examples for intersectoral costs and benefits (ICBs) resulting from healthcare interventions that affect the education sector could include special education services and the costs for student transport to the education facility [56, 57]. Criminal justice inter-sectoral costs compromise costs for court proceedings, police services, or forensic services [56, 58]. The methodology for the valuation of health-related service use in the education and (criminal) justice sectors is less established. A first major step towards the valuation of such service use was recently taken by determining several methods and testing their applicability in the Netherlands [59]. The four methods suggested for consideration based on their accuracy in a hierarchical manner include i) the opportunity cost method based on micro-costing, ii) utilisation of market prices from governmental reports, iii) self-constructed unit prices based on the information given in governmental reports, and iv) hourly labour costs for the provision of the relevant services. Although the opportunity cost method based on micro-costing (method i) is very accurate in regards to the valuation of ICBs in monetary terms, its feasibility is limited by the time it takes to calculate these [13]. Method ii) can be applied to determine proxy unit prices. Examples for relevant sources include annual reports of governmental and public or private organizations, which have been granted authority and responsibility for the provision of services related to ICBs. The reliability, transparency, unrestricted availability and unambiguous referencing are a prerequisite for the validity of this method [13]. The third method (iii) suggests using the previously mentioned annual reports for the division of the total annual costs by the total annual output. Labour costs can be used as a fourth method for valuation (method iv), e.g. from national statistics and administrative data [13]. The methodological choice should be based on the availability and reliability of the underlying data [13].

### Country specific requirements and recommendations

A summary of some key costing recommendations from national health economic guidelines of the selected six countries is shown in Table 3 (extended structured synthesis Additional file 1). While these guidelines are defined as being mandatory to be followed in the Netherlands and Germany, the Spanish guideline is voluntary. The application of the Austrian, English and Hungarian guidelines is (strongly) recommended. This does not necessarily imply, however, that recommendations are also relevant for the pharmaceutical reimbursement process as in the case of Austria, where the relevant institution applies its internal criteria [60].

**Table 3:** Overview of country-specific costing recommendations in the six selected European countries

	**Austria**	**England**	**Germany**	**Hungary**	**The Netherlands**	**Spain**
**Recommended level of guideline adherence**						
Mandatory			x^a^		x	
Recommended	x	x		x^b^		
Voluntary						x
**Perspective**						
Societal					x	
Societal and health care				(x)^c^		x
Health and social care		x				
Health care			x			
Not specified	x					
**Discounting recommended**	x	x	x	x	x	x
Discount rates costs (sensitivity analysis)	5% (3%-10%)	3.5% (1.5%)	3% (0%, 5%)	3.7% (2%-5%)	4% (not specified)	3% (0%, 5%)
**Reporting of costs**						
Separate reporting	x	x	x	x^d^	x	
Not explicitly mentioned						x
**Adjustment for inflation/price indexation**						
Adjustment to a common reference year	x			x		x
Inflation of costs to the present			x		x	
No information found		x				
**Time horizon**						
A lifelong time horizon					x	x
Long enough to include all costs/outcomes/effects		x	x	x	x	x
No information found	x					
**Periodicity of costing manual**						
Annually		x				
Regularly	x			x		
As required for methodological reasons (approx. every 4 year)					x	
Irregularly			x			
No information found						x

^a^The recommendations stated in the German guidelines by IQWIG (Institute for Quality and Efficiency in Health Care) are only binding for the IQWIG itself

^b^The application of the guideline is defined not by the user, but by the purpose of the analysis. The guideline applies to the assessment of all health technologies for decision making in public funding

^c^Specification in the Hungarian guideline: The recommended perspective is the healthcare perspective. However, if the benefits and costs are to a significant extent outside of the health care system (e.g. in the case of preventive health technologies), it is also suggested to add a social perspective to the analysis

^d^Cost and outcome results for the entire time horizon of the analysis should also be presented separately by health status and presented in tabular form

With regards to the adopted analytical study perspective of an EE, the German and the English recommend a healthcare perspective. The Austrian guideline is not specific in this regard. While the Dutch guideline recommends a societal perspective, the Spanish one recommends both the societal and the healthcare perspectives to be applied parallel. The Hungarian guideline states that if costs and outcomes are falling mainly outside of the healthcare system (e.g. in the case of preventive health technologies) the societal perspective is suggested in addition to the healthcare perspective. That what is meant by societal is, however, is not always explicitly stated and may vary very much from analysis to analysis.

Most guidelines state which costs should be included in an EE. The majority of them use a traditional cost component typology referring to health and social care and out-of-pocket expenses as direct costs and lost productivity as indirect costs [61]. The Dutch guideline, updated in 2016, is an exception and refers to cost components based on sectors, such as costs within the healthcare sector, patient and family costs and costs in other sectors [62].

The inclusion of social care costs such as those resulting from the use of respite care and supportive care services is explicitly recommended in four guidelines (England, Germany, the Netherlands, Spain), while the inclusion of patient (and family) costs (e.g. patient out-of-pocket expenses, time costs, informal care costs and travel costs) are recommended in four guidelines (Austria, Germany, the Netherlands, Spain). In Germany, patients’ out-of-pocket expenses are included as part of the perspective of the Statutory Health Insurance (SHI)-insured community. In contrast, time costs are not standardly considered, but are examined in the course of sensitivity analyses. The Hungarian guideline specifies that the inclusion of cost types should be determined based on the adopted perspective. The inclusion of lost productivity costs is recommended in four guidelines (Austria, Germany, the Netherlands, Spain) and are to be reported separately from direct medical costs). In the Hungarian guideline the inclusion of productivity costs is recommended just a complementary element of cost calculation if majority of costs are falling outside of the healthcare sector.

There is large variation between the guidelines regarding the suggested valuation approach. All guidelines stress that the valuation method chosen should reflect opportunity costs. In addition, various valuation methods are recommended in the guidelines, varying from top-down micro-costing to gross-costing, including the use of standard unit costs/reference prices, tariffs, market prices, administrative data, and diagnosis-related groups (DRGs). All guidelines name one or more sources, which should preferably be used for the unit costing. A standard cost list containing sets of standardized unit cost estimates is officially recognized and applied by the HTA agency in the Netherlands (Zorginstituut Nederland) [63]. These have been (partly) published as part of national costing guidelines/programs and are periodically updated. In Spain, each region has its own list of official unit costs [64]. There are also national statistics for some of the unit costs (e.g. hospitalization) managed by the ministry of health [65]. In addition, the unit cost database OBILUKE is used in Spain to obtain healthcare unit costs. It is updated annually and uses primary and secondary sources, such as published articles, reports, hospital accounting systems, but access to this unit cost database is not free of charge [66]. Other countries (e.g. Germany) also have unit cost projects in place that have been conducted systematically with a clear methodology, but are not regularly updated. In Austria, a list with unit costs used in existing Austrian health economic analyses has been published [48, 67]. Updates of the national unit cost lists are published periodically spanning from annual updates (e.g. England and Wales) to updates as required for methodological reasons or timeliness, e.g. four-yearly in the Netherlands.

There is limited information in the majority of the guidelines about the valuation and allocation of capital/overhead and operating costs, i.e. costs that cannot be directly allocated. Austria and Spain do not include recommendations on this at all. The Dutch guideline names different methods that can be used to measure overheads such as average fixed costs per unit, the equivalence method, and the mark-up method. Although no specific method is recommended, it is emphasized that each of these methods has different advantages and disadvantages. Based on data from the financial statistics for Dutch hospitals, an estimation of overhead costs was conducted in 2012. The derived percentage for overheads on the directly attributable costs of medical departments was 38%. The percentage for overheads on housing and depreciation costs on the directly attributable costs of medical departments was 6%, adding up to a total of 44% for overheads. This percentage reflects an average with huge variations between individual organisations and services [63].

For the adjustment of unit costs from different calendar years, the Austrian and Spanish guidelines recommend that costs should be adjusted to a common reference year, but they fail to provide further details. The Hungarian guideline states that costs shall be uprated to the same date. The consumer price index (inflation) should be chosen as inflation rate, irrespectively of whether the costs (or savings) arise within or outside the healthcare sector, with the official publications of the Hungarian Central Statistical Office as recommended source. Both the German and Dutch guidelines specify that all costs should be inflated to the present value by using the official price index from national statistics. The Dutch guideline also tackles the aspect of correcting for inflation in case of including costs from different European countries. It is suggested that the Indices of Consumer Prices (HICP) of the European Central Bank, which were specially designed for international comparisons of consumer price inflation, need to be used.

## Discussion

For EEs to be able to inform efficient resource allocation based on valid high-quality evidence, it is crucial that both outcomes and costs are assessed rigorously [68]. In contrast to outcomes, costs appear to have suffered neglect regarding methodological research, resulting in the absence of an universally accepted costing methodology for the healthcare sector [69], as well as other sectors affected by the impact of healthcare interventions such as the education and criminal justice sectors [13]. Unit cost estimates between studies and countries are often not comparable due to differences in costing methodologies [70] and the lack of detailed methodological guidance, which may also result in decision-makers’ low confidence as a barrier to the uptake of EEs [43, 71]. As of now, collections of cost estimates are not routinely available for services across European countries, especially beyond the health and social care sectors [72]. If available, it is unclear whether differences in cost estimates stem from differences in the service composition, intensity and definition (unit of analysis), differences in the unit of measurement, or differences in methodological approaches for costing including input costs (unit of valuation) [73], calling for more transparency and harmonization in this respect.

This scoping review highlights multiple methodological problems challenging the harmonization of service unit costs for (inter)national, multi-sectoral health EEs. For the health and social care sectors, these include ambiguity and feasibility problems regarding the definition and application of cost identification and valuation approaches. Several problems regarding the data source used to develop unit costs were identified: the impact of choice of the costing sources, the applied costing perspective and unavailable proxy measures, as well as the absence of a standard to estimate overhead costs.

Moreover, the limited cross-country comparability of labour costs and ambiguity concerning the application of cost types were identified as further difficulties. Challenges in respect to the education and criminal justice sectors concern the valuation of health-related service use in these sectors, unestablished valuation methods, and missing feasibility testing in the international context, as well as difficulties regarding the generation of proxy unit prices. Double counting of costing components also may not only be an issue within the health care sector as previously reported, but also cross-sectorial. For example, double counting of out-of-pocket expenses may occur as part of the unit cost of a health care service and as part of patients’ out-of-pocket costs, which may arise due to lacking transparency in the reported costing components. The analysis of national costing guidelines revealed extensive variation concerning the recommendations with respect to guideline adherence, analytical study perspective, valuation approach and inflation.

The early FP6-funded HealthBasket project (2004–2007), which focused on harmonization of costing in the healthcare sector, already concluded one decade ago that “the prerequisite for international cost comparison is mutually accepted methodological guidance (standard costing method) and reasonably good compliance with it” [22]. Furthermore, it stated that consensus alone on basic scientific principles would not be sufficient to achieve meaningful comparability. Instead, it was proposed to “standardize” the most important and frequently used methods, including resource use measurement, cost allocation methods, including allocation base and allocation techniques and valuation methods, as well as capacity utilisation and to include detailed instructions on how to implement these instruments in practice [22]. Another recent editorial also recommended that an independent group should be mandated with the production of standard country-specific unit costs available to national and international researchers and decision-makers [70]. These challenges were one of the key motivations for setting-up the European PECUNIA project, aiming among others to systematically address the above heterogeneity-causing factors in costing methods and to develop unit costs for different countries and sectors based on harmonized methods [18, 74].

At the same time, some practical limits to the harmonization of costing will remain. Firstly, unit costs are one major ingredient to the valuation aspect in EEs. Limited guidance regarding the calculation of overheads resulting in assumptions are a very much under-discussed, under-researched and under-reported area. Both, extensive sensitivity analysis or transparent overhead calculation as one harmonization aspect for unit costs would be potential ways forward. There are, however, also broader costing issues [45], such as questions about the inclusion of future medical costs [75], the choice of the discount rate [40], and the choice of the analytical study perspective [76] that may introduce systematic differences in cost estimates [77, 78]. Secondly, where newly developed harmonization strategies are not fully in line with existing national EE guidelines, especially those relevant for reimbursement decisions, their implementation most likely will face resistance. The quality and transferability of international EE studies that include multiple countries would, however, still greatly benefit from such standardisation approaches [40]. The same applies to national EEs where such perceived conflict does not exist, or the evaluation perspective is expected to be expanded to a multi-sectoral, societal one. On the other hand, the compulsory nature of EE guidelines seem to promote the availability of more comprehensive and standardised unit cost catalogues (e.g. England, Netherlands) and the use of more harmonised costing methods.

To the best of our knowledge, this is the first review on costing methods across several sectors affected by healthcare interventions focusing on a set of selected European countries and healthcare systems. Other publications focusing on differences between EE guidelines do exist (e.g. [79, 80]), but they cover different aspects (e.g. methods for price and currency adjustment, uncertainty analysis), additional countries outside of Europe (e.g. Australia, Thailand, Japan), or include older guideline versions.

The selected countries included in the PECUNIA Project differ regarding their health care systems with varying feasibility and acceptability of EEs in evidence-informed decision-making. Some countries have established national unit cost programmes/lists (DE, NL, UK), some early stage initiatives (AT, ES, HU). Availabilities of health utility value sets for outcome evaluations and requirements in terms of the study perspective also differ. Nevertheless, the country selection is not necessarily balanced in regards to the aforementioned aspects.

Study selection and data extraction was challenging in some cases, especially when slightly divergent information on one topic was identified. However, two researchers were involved in the study selection process and data extraction phase, and with two additional researchers consulted in case of disagreements, which enabled extensive discussions and thorough assessment of the identified material. Due to the broad topic area and expected spread of relevant information between different types of publications, mostly within the grey literature needed to be included. Our search strategy to identify grey literature was very comprehensive and national experts were involved to complement our findings. In addition, a search update was conducted to enhance the review’s timeliness. Although the applied strategy to identify peer-reviewed publications was expected to capture all relevant key topics, as it covered different approaches which were explained in our methods section, it cannot be ruled out that some potentially relevant material has been left out, despite our attempts to be as comprehensive as possible.

## Conclusions

Several methodological issues were identified that lead to the current heterogeneity in valuation methods in health EEs used across sectors and countries. To address these explicitly in future costing guidelines and tools is a key step towards more comparability and harmonization in national and international health EEs. 

## Supplementary Information

**Additional file 1:** **Figure 1.** Adapted PRISMA flow chart of study selection. **Table 2.** Preferred Reporting Items for Systematic reviews and Meta-Analyses extension for Scoping Reviews (PRISMA-ScR) Checklist. **Table 3.** Synthesis of country-specific recommendations regarding valuation aspects from economic evaluation guidelines of six selected European countries.

## Data Availability

All data generated or analysed during this study are included in this published article.

## Abbreviations

BIA: Budget impact analysis
Catsalut: The Catalan Health Service (Servei Català de la Salut)
CBS: Statistics Netherlands (Centraal Bureau voor de Statistiek)
CPI: The consumer price index
DoH: Department of Health
DRGs: Diagnose-related groups
EE(s): Economic evaluation(s)
EU: European Union
EQUATOR: The Enhancing the QUAlity and Transparency Of health Research Network
FCA: The friction cost approach
FTE: Full Time Equivalent
HBCs: Diagnosis Related Group, DRG (homogen betegsegcsoport)
HCA: The human capital approach
HES: Hospital Episode Statistic
HICP: The Indices of Consumer Prices
HRG: Healthcare Resource Group
HTA: Health Technology Assessment
ICBs: Inter-sectoral costs and benefits
iHEA: The international Health Economics Association
ISPOR: The International Society for Pharmacoeconomics and Outcomes Research
IQWIG: The independent Institute for Quality and Efficiency in Health Care (Institut für Qualität und Wirtschaftlichkeit im Gesundheitswesen)
n/a: Not available
NHS: National Health Service
NZA: Dutch healthcare authority (Nederlands Zorg Autoriteit)
OENO: International Classification of Procedures in Medicine
OSTEBA: Basque Office for Health Technology Assessment
PPP: Purchasing power parities
PSS: Personal social services
PECUNIA: The European ProgrammE in Costing, resource use measurement and outcome valuation for Use in multi-sectoral National and International health economic evaluAtions
PSSRU: The Personal Social Services Research Unit
RRISMA-ScR: Preferred Reporting Items for Systematic reviews and Meta-Analyses extension for Scoping Reviews
SA: Sensitivity analysis
SHI: Statutory Health Insurance
UKE: The University Medical Center Hamburg-Eppendorf
VAT: Value-added tax
