# Supplementary material for: Harmonization issues in unit costing of service use for multi-country, multi-sectoral health economic evaluations: a scoping review
Source: Health Econ Rev. 2022 Aug 3;12:42. doi: 10.1186/s13561-022-00390-y (PMC9347135; doi:10.1186/s13561-022-00390-y)
Supplement: Supplementary file 1 — Additional file 1: Figure 1. Adapted PRISMA flow chart of study selection. Table 2. Preferred Reporting Items for Systematic reviews and Meta-Analyses extension for Scoping Reviews (PRISMA-ScR) Checklist. Table 3. Synthesis of country-specific recommendations regarding valuation aspects from economic evaluation guidelines of six selected European countries. [file 13561_2022_390_MOESM1_ESM.docx]

**Additional file 1**

**Additional file Figure 1.** Adapted PRISMA flow chart of study selection


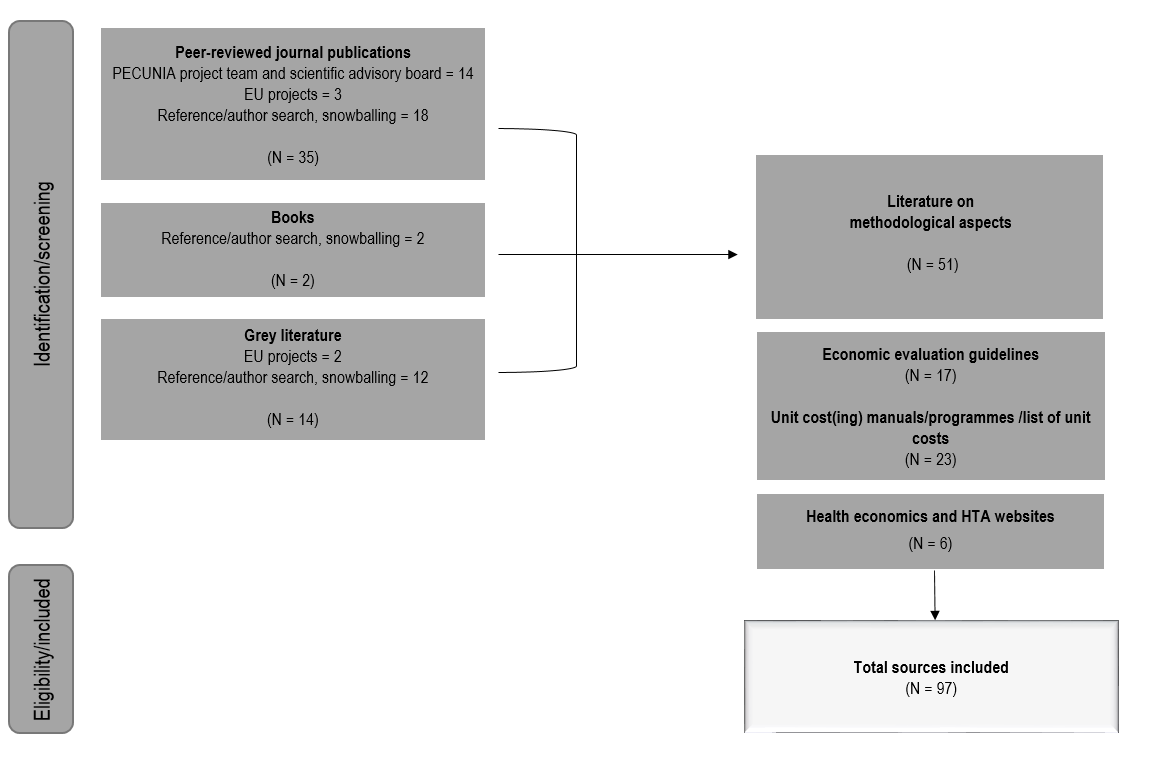


**Additional file Table 2: Preferred Reporting Items for Systematic reviews and Meta-Analyses extension for Scoping Reviews (PRISMA-ScR) Checklist**

| SECTION | ITEM | PRISMA-ScR CHECKLIST ITEM | REPORTED ON PAGE # |
| --- | --- | --- | --- |
| TITLE | | | |
| Title | 1 | Identify the report as a scoping review. | 1 |
| ABSTRACT | | | |
| Structured summary | 2 | Provide a structured summary that includes (as applicable): background, objectives, eligibility criteria, sources of evidence, charting methods, results, and conclusions that relate to the review questions and objectives. | 3 |
| INTRODUCTION | | | |
| Rationale | 3 | Describe the rationale for the review in the context of what is already known. Explain why the review questions/objectives lend themselves to a scoping review approach. | 5 |
| Objectives | 4 | Provide an explicit statement of the questions and objectives being addressed with reference to their key elements (e.g., population or participants, concepts, and context) or other relevant key elements used to conceptualize the review questions and/or objectives. | 6/7 |
| METHODS | | | |
| Protocol and registration | 5 | Indicate whether a review protocol exists; state if and where it can be accessed (e.g., a Web address); and if available, provide registration information, including the registration number. | 7 |
| Eligibility criteria | 6 | Specify characteristics of the sources of evidence used as eligibility criteria (e.g., years considered, language, and publication status), and provide a rationale. | 7/8 |
| Information sources* | 7 | Describe all information sources in the search (e.g., databases with dates of coverage and contact with authors to identify additional sources), as well as the date the most recent search was executed. | 7/8 |
| Search | 8 | Present the full electronic search strategy for at least 1 database, including any limits used, such that it could be repeated. | 7 |
| Selection of sources of evidence† | 9 | State the process for selecting sources of evidence (i.e., screening and eligibility) included in the scoping review. | 7/8 |
| Data charting process‡ | 10 | Describe the methods of charting data from the included sources of evidence (e.g., calibrated forms or forms that have been tested by the team before their use, and whether data charting was done independently or in duplicate) and any processes for obtaining and confirming data from investigators. | 8 |
| Data items | 11 | List and define all variables for which data were sought and any assumptions and simplifications made. | 8 |
| Critical appraisal of individual sources of evidence | 12 | If done, provide a rationale for conducting a critical appraisal of included sources of evidence; describe the methods used and how this information was used in any data synthesis (if appropriate). | n.a. |
| Synthesis of results | 13 | Describe the methods of handling and summarizing the data that were charted. | 8 |
| RESULTS | | | |
| Selection of sources of evidence | 14 | Give numbers of sources of evidence screened, assessed for eligibility, and included in the review, with reasons for exclusions at each stage, ideally using a flow diagram. | 9 / Figure 1 |
| Characteristics of sources of evidence | 15 | For each source of evidence, present characteristics for which data were charted and provide the citations. | Appendix |
| Critical appraisal within sources of evidence | 16 | If done, present data on critical appraisal of included sources of evidence (see item 12). | n.a. |
| Results of individual sources of evidence | 17 | For each included source of evidence, present the relevant data that were charted that relate to the review questions and objectives. | Appendix |
| Synthesis of results | 18 | Summarize and/or present the charting results as they relate to the review questions and objectives. | 9 - 14 |
| DISCUSSION | | | |
| Summary of evidence | 19 | Summarize the main results (including an overview of concepts, themes, and types of evidence available), link to the review questions and objectives, and consider the relevance to key groups. | 15 / 16 |
| Limitations | 20 | Discuss the limitations of the scoping review process. | 16 |
| Conclusions | 21 | Provide a general interpretation of the results with respect to the review questions and objectives, as well as potential implications and/or next steps. | 17 |
| FUNDING | | | |
| Funding | 22 | Describe sources of funding for the included sources of evidence, as well as sources of funding for the scoping review. Describe the role of the funders of the scoping review. | 17 |

JBI = Joanna Briggs Institute; PRISMA-ScR = Preferred Reporting Items for Systematic reviews and Meta-Analyses extension for Scoping Reviews.

* Where *sources of evidence* (see second footnote) are compiled from, such as bibliographic databases, social media platforms, and Web sites.

† A more inclusive/heterogeneous term used to account for the different types of evidence or data sources (e.g., quantitative and/or qualitative research, expert opinion, and policy documents) that may be eligible in a scoping review as opposed to only studies. This is not to be confused with *information sources* (see first footnote).

‡ The frameworks by Arksey and O’Malley (6) and Levac and colleagues (7) and the JBI guidance (4, 5) refer to the process of data extraction in a scoping review as data charting*.*

§ The process of systematically examining research evidence to assess its validity, results, and relevance before using it to inform a decision. This term is used for items 12 and 19 instead of "risk of bias" (which is more applicable to systematic reviews of interventions) to include and acknowledge the various sources of evidence that may be used in a scoping review (e.g., quantitative and/or qualitative research, expert opinion, and policy document).

*From:* Tricco AC, Lillie E, Zarin W, O'Brien KK, Colquhoun H, Levac D, et al. PRISMA Extension for Scoping Reviews (PRISMAScR): Checklist and Explanation. Ann Intern Med. 2018;169:467–473. [doi: 10.7326/M18-0850](http://annals.org/aim/fullarticle/2700389/prisma-extension-scoping-reviews-prisma-scr-checklist-explanation).

**Additional file Table 3: Synthesis of country-specific recommendations regarding valuation aspects from economic evaluation guidelines of six selected European countries**

|  | Austria | England | Germany | Hungary | The Netherlands | Spain |
| --- | --- | --- | --- | --- | --- | --- |
| REFERENCES |  | | | | | |
| Economic evaluation/Health Technology Assessment (HTA) guidelines and manuals (year of publication) | (81, 82) – latest versions (2012, 2006); German version (83) (2006) | England**:  Gold standard: (84) (2013)  Diagnostics technologies: (85) (2011)  (Resource impact: (86) (2017)) | (87)– latest version (2020)  (88, 89) – previous versions (2017, 2009) | (90)– latest version (2021)  (91) – previous version (2017) | Health and social care: (92)  ICB: (13) | Basque Country (and Spain):  (93)– latest version (2010)  (Spanish version (94)) (2010)  Andalusia: (95) (2015)  (Catalonia: (96) (2014)) |
| Available costing programme/manual/guideline | Database of published unit cost estimates: (48)  Unit cost research project based on unit costs published in the literature (67) | England: (97-99) | Costing references for health and social care: (100-108)  No list of unit costs (but studies - Bock et al. (106). | Not available | Health and social care: (62, 63) – latest versions  (109, 110) – previous versions  ICB*: (13, 111) | Catalonia:  Health care: (112, 113)  eSalud unit cost listing by Obiluke Consulting (private company) (66)  Yes, Bendeck et al. 2013; Generalitat de Catalunya, 2010. |
| ISPOR* profile | (114) | England/Wales: (115) | (116) | (117) | (118) | (119) |
| Recommended resource use measurement (RUM) instrument | Not available | Not available | Not available | Not available | Employment/productivity: (120, 121)  Informal care: (122) | Not available |
| METHODOLOGICAL ISSUES |  | | | | | |
| Perspective | Choice of perspective must derive logically from research question and be justified;  Results to be presented separately for different perspectives; | Healthcare, social care | Healthcare (usually statutory health insurant).  (If loss of productivity is substantially affected by new health technology, corresponding costs may be evaluated separately.) | If costs and outcomes are falling mainly outside of the healthcare system the societal perspective is recommended in addition to the healthcare perspective | Societal perspective | Societal perspective and healthcare perspective |
| Costs  (to be included) | Healthcare, patient, productivity | Healthcare, social care | Healthcare, patient, social care, separate reporting in SA*: productivity; Unrelated future costs, implementation costs | Should be determined based on the perspective. Health insurance: direct costs. Societal: all costs incurred in the society.  Inclusion of productivity costs is recommended just a complementary element of cost calculation if majority of costs are falling outside of healthcare sector. In that case, productivity costs must be reported separately.  The guideline allows some flexibility in special cases with justification. | Healthcare, social care, patient and family, criminal justice, education, education, productivity | Healthcare, social care, patient and family, productivity |
| Source of valuation  (cost data) | Opportunity costs / market prices → payer tariffs → shadow prices (82) | Current official listing published by the DoH* and/or the Welsh Assembly Government | Statutory health insurance, further considerations depending on perspective chosen | Recommendation to use the official OENO* codes (<http://finanszirozas.oep.hu/szabalykonyv/index.asp?mid=1>)  and HBCs* listings (these are the DRG* points to the above OENO* codes: <http://www.oep.hu/felso_menu/szakmai_oldalak/gyogyito_megeleozo_ellatas/szabalykonyvek/besorolo.html>) (91) | Important sources for rating units are 1) reference prices; 2) own cost price research; 3) financial records, Nza* rates, market prices, national registrations or already published literature.  If in the absence of national cost data, data from international publications are used, these must be validated for the Dutch setting. One way to accomplish this is to request input from an expert panel. | Official publications, accounts of healthcare centres, and the fees applied to NHS service provision contracts. |
| Hierarchy of data sources stated for valuation (cost evidence) used // Measurement of resource use | 1) Austrian cost studies based on cost accounting data;  2) Meta tariff system or mixed tariffs from several payer tariff catalogues, or tariff catalogue of one regional health insurance fund;  3) All service and price catalogues of the social insurance agencies, hospitals, nursing homes, rehabilitation centres, geriatric centres, sanatoria, Medical Chamber, pharmacists´ professional association;  4) Tariff catalogue of the Austrian DRG* system for fund hospitals;  5) Data from cost calculations of hospitals;  6) Estimates from Delphi panels;  7) Empirical surveys;  8) Expert opinion.  (82).  All data sources used must be described in detail, their selection must be justified, their suitability and validity assessed. In doing so, the internal as well as the external validity shall be considered. // Patient level, primary level, secondary level | The sources that best reflect the price relevant to the NHS* are preferred (for the Technology Appraisal Programme: public list prices, nationally available price reductions, prices paid for some generic drugs, patient access scheme, national average unit cost of an HRG*). Data based on HRGs* may not be appropriate in all circumstances (for example, when the new technology and the comparator both fall under the same HRG*, or when the mean cost does not reflect resource use in relation to the new technology under appraisal) and other sources of evidence, such as micro-costing studies, tariff or unit costs, may be preferred. // n/a* | Recent studies from literature → own calculations from SHI* routinely collected data → guidelines → expert opinion only if data availability very limited. // Patient level, primary level. | In the case of studies which adapt resource use data from foreign clinical studies or health EEs clinical practice in the foreign setting should be compared (and recalculated) with the Hungarian one. // Patient level, primary level. | See section ‘Sources of valuation’: 1) reference prices 2) own cost price research 3) financial records, NZA* rates, market prices, national registrations or already published literature. // Patient level, primary level. | Costs should be evaluated based on opportunity cost (i.e., the best available alternative) (Spanish recommendations, CATSALUT, and OSTEBA). Due to imperfections on the healthcare market, it is probably more useful to rely on official publications, accounts of healthcare centers, and the fees applied to NHS* service provision contracts. Non-health-care costs should be identified individually and in detail using surveys designed for this purpose (Spanish recommendations). // Patient level, primary level. |
| Costing methodology (valuation of costs) | Micro-costing. If not feasible, macro-costing approach/gross-costing must be justified. Alternatively, use of unit costs (average costs).  Productivity loss: HCA* preferred; use of FCA* must be justified. | Tariffs, reference costs or unit prices from NHS* organizations. (Costs should relate to NHS* and PSS* resources and should be valued using the prices relevant to the NHS* and PSS*) | Macro-costing, micro-costing, DRG*, FCA* | Tariffs, lowest price, include VAT* and also DRG* for inpatient care and some outpatient care activities | If possible, bottom-up micro-costing to calculate reference prices (gold standard for calculating cost prices). When bottom-up micro-costing data not available, gross costing methods were applied to calculate reference prices. FCA*. | Opportunity costs, unit costs, paid employment-labour production losses (measured using the HCA* or FCA*), unpaid work-housework and Informal care time (measured based on opportunity costs, replacement cost, or declared preference method), and leisure time (based on shadow costs as a proxy to opportunity costs). |
| Measurement of capital costs, overhead costs and operating costs | - | Different methods: e.g. % on fees or FTE* allocation. In instances where capital costs are differentiated (buildings and oncosts, land and other capital) mostly no additional overheads added.  Sources: financial accounts. | Capital costs for inpatient services: For each inpatient day based on capital expenditure per bed.  Operational costs: Division of hospitals expenditures of all funding bodies by bed occupancy rate. | Gross salaries to be used at all times (no further recommendation in (91)).  (In practice, hourly rates from statistical data are calculated.) | Different methods that can be used to measure overheads: average fixed costs per unit, the mark-up method, and equivalence method. No specific method is recommended, however, emphasized that each of these methods has advantages and disadvantages. | - |
| Physical units (e.g. hours of physician/nursing; per visit; work-day) presented? / Any common units used? | n/a* | Stay, client/patient hour, visit, consultation, resident week, episode, day, procedure, attendance, session | Per contact, patient, inpatient day, (contact) minutes, care day, rehabilitation day, package, treatment unit, hour, person, year, employed, (direct) contact, hour, conversation, day nursery place per year) | Depending on the service, as they are financed differently (see row above).  Daily rate: rehabilitation, long-term care, etc.  DRG* codes are often used for inpatient care.  Hourly rate and number of visits are also used, but it varies a lot from analysis to analysis. The guideline does not specify this. | Per day: inpatient, ICU, inpatient elderly, day care treatment, healthcare for disabled patients per visit: outpatient, ER, GP, paramedical care, mental healthcare per hour: home care, rehab therapy | Studies: per event, visit inpatient stay |
| Reporting of costs (any requirement to report costs and quantities/volumes separately?) | Separate reporting. | Separate reporting. | Separate reporting. | Not explicitly mentioned. | Separate reporting. | Not explicitly mentioned, but recommended to describe data separately. |
| Time horizon | Not available. | Long enough to reflect all important differences in costs/outcomes between the technologies being compared. | Must at least represent the average study duration and thus consider the differences in cnb* for the decision. Particular for chronic diseases, a longer time horizon should be chosen if possible. Decision-analytic models are often applied for health EEs over longer time horizons. Costs and benefits should always be modelled over the same time horizon.  The appropriate time horizon is often longer than the period covered by the available primary data from prospective studies. In these cases, , a time horizon appropriate for the disease should be chosen | Long enough to include all costs and effects, but adjust to Hungarian life expectancy (91). (In practice, 10 years are the most common time horizon). | Preferably there should be a lifelong time horizon. One can choose a different time horizon if the lifelong one is not appropriate. In that case the time horizon of an EE* should enable making a valid and reliable conclusion on the differences between effectiveness and costs of the compared interventions. | Should capture all relevant differences in costs and in the effects of health treatments and resources. In some cases, time horizon will have to be extended to the individual’s entire life. |
| Discounting | 5% discount rate.  SA*: higher and lower rates (e.g. 3% and 10%). | 3.5% discount rate.  SA*: 1.5%. | 3% discount rate.  SA*: 0%, 5%. | 3.7% discount rate.  SA*: 2-5%. | 4% discount rate.  SA*: not specified. | 3% discount rate.  SA*: 0% and 5%. |
| Adjustment for inflation/price indexation  How updating of costs to the relevant year and currency should be done | Costs should be adjusted to reference year and converted into Euro using PPP*. | Not available. | Costs from different time periods should be adjusted. General price index (published by the Federal Statistical Office) is used therefore. | Prices shall be converted to the same date (possibly present date). CPI* (inflation) should be chosen as conversion rate, irrespectively of where the costs (or savings) arise, within or outside the healthcare sector. The official publications of the Hungarian Central Statistical Office should be consulted on annual price index. | All costs should be converted into present value using Dutch Statistics Bureau price index. In the event that cost prices of different units or volumes are not from the same calendar year, the prices should be corrected for inflation between the years. This is done with the use of the CPI*, available in the CBS* StatLine database | Costs should be adjusted to reference year (OSTEBA* and CatSalut*). |
| Specific considerations per field/area of application | No restrictions. | Health technologies and their selection: medicinal products medical devices, diagnostic techniques, surgical procedures,  therapeutic technologies other than medicinal products, systems of care, screening tools. | Medical interventions. | Guideline was specifically designed for pharmaceutical products, but the guideline allows some flexibility due to e.g. the increasing number of reimbursement submissions for medical devices. | Reference case analysis was framed in particular for EEs in the fields of pharmaceutical care and curative care. - Reference case: All costs in the healthcare system, patient/family, and other sectors. Productivity losses: FCA. - Prevention: follow as strictly as possible for sake of uniformity and comparability --> Extra attention needed for intersectoral costs;  - Diagnostics: follow as strictly as possible,  - Medical devices: follow as strictly as possible --> Utilization degree influences the fixed price per unit and thus the cost price (such as depreciation charges). Collective devices: employable in more than one population has cost-reducing effect on the price per procedure (synergy gains);  - Long-term care: follow at minimum - Forensics: best to follow --> Quantify costs for ’patient’ and ‘victim’. | Health technologies, no other areas specified. |
| Periodicity of costing manual | EE* guideline: 2006, 2012 Costing: DHE* Unit Cost Database updated regularly (approx. every 2 years). | Annually. | EE guideline: irregular;  Costing (UKE*): no info. | The EE* guideline is regularly updated, but it is mainly an activity of the Hungarian Health Economic Association (META).  The National Health Insurer provides updates when needed. | When required for methodological reasons (approx. every 4 years). | n/a* |
| Recommended level of guideline adherence | Recommended | Recommended | Mandatory | Strongly recommended | Mandatory | Voluntary |

* BIA – Budget impact analysis; catsalut – The Catalan Health Service; CBS - Statistics Netherlands (Centraal Bureau voor de Statistiek); cnb – costs and benefits; CPI – the consumer price index;DHE – department of health economics; DoH – Department of Health; DRG – Diagnosis Related Group; EE(s) – economic evaluation(s); FCA – the friction cost approach; FTE – Full Time Equivalent; HBCs - Diagnosis Related Group, DRG (homogen betegsegcsoport); HCA - the human capital approach; HES – Hospital Episode Statistic; HRG – Healthcare Resource Group; ICB – inter-sectoral costs and benefits, ISPOR - the International Society for Pharmacoeconomics and Outcomes Research; n.a. – not available; NHS – National Health Service; NZA - Nederlands Zorg Autoriteit - Dutch healthcare authority, OENO - International Classification of Procedures in Medicine (orvosi eljarasok nemzetkozi osztalyozasa), OSTEBA – Basque Office for Health Technology Assessment; PPP – purchasing power parities; PSS – personal social services; PSSRU - the Personal Social Services Research Unit; SA – sensitivity analysis; SHI – Statutory Health Insurance; UKE – the University Medical Center Hamburg-Eppendorf (Universitätsklinikum Hamburg-Eppendorf); VAT – value-added tax.

** Guidance produced by the Institute on health technologies is also applied selectively in Northern Ireland, Scotland and Wales. (84)

**References**

1. LBI HTA. Methodenhandbuch für Health Technology Assessment Version 1 2012. <https://hta.lbg.ac.at/uploads/tableTool/UllCmsPage/gallery/Methodenhandbuch.pdf>. Accessed 10 May 2020.
2. Walter E, Zehetmayr S. Guidelines zur gesundheitsökonomischen Evaluation Konsenspapier. Wien Med Wochenschr. 2006;156(23-24):628-32.
3. Walter E, Zehetmayr S. Theoretische Implikationen zur gesundheitsökonomischen Evaluation mit Ausblick auf Österreich. Wien Med Wochenschr. 2006;156(23-24):622-7.
4. NICE – National Institute for Health and Care Excellence. Guide to the methods of technology appraisal 2013 (PMG9) 2013 [updated 2018]. <https://www.nice.org.uk/process/pmg9/resources/guide-to-the-methods-of-technology-appraisal-2013-pdf-2007975843781>. Accessed 10 May 2020.
5. NICE - National Institute for Health and Care Excellence. Diagnostics Assessment Programme manual, December 2011: NHS - National Health Service; 2011. <https://www.nice.org.uk/Media/Default/About/what-we-do/NICE-guidance/NICE-diagnostics-guidance/Diagnostics-assessment-programme-manual.pdf>. Accessed 10 May 2020.
6. NICE - National Institute for Health and Care Excellence. Assessing resource impact process manual: guidelines: NHS - National Health Service; 2017. <https://www.nice.org.uk/Media/Default/About/what-we-do/Into-practice/RIA-process-manual-guidelines.pdf>. Accessed 10 May 2020.
7. IQWiG – Institute for Quality and Efficiency in Health Care. Allgemeine Methoden (Version 6.0). 2020.
8. IQWiG – Institut für Qualität und Wirtschaftlichkeit im Gesundheitswesen. Allgemeine Methoden, Version 5.0 vom 10.07.2017. Köln: IQWiG (Institute for Quality and Efficiency in Health Care); 2017. <https://www.iqwig.de/download/Allgemeine-Methoden_Version-5-0.pdf>. Accessed 10 May 2020.
9. IQWiG – Institut für Qualität und Wirtschaftlichkeit im Gesundheitswesen. Arbeitspaper Kostenbestimmung - Version 1.0 vom 12. Oktober 2009. 2009. <https://www.iqwig.de/download/Arbeitspapier_Kostenbestimmung_v_1_0.pdf>. Accessed 10 May 2020.
10. Ministry of Human Resources. Az Emberi Erőforrások Minisztériuma egészségügyi szakmai irányelve az egészség-gazdaságtani elemzések készítéséhez és értékeléséhez [Guidelines of the Ministry of Human Resources on the methodology of health economic evaluation]. Egészségügyi Közlöny. 2021;71(21):2178–2200.
11. Ministry of Human Resources. Az Emberi Eroforrások Minisztériuma szakmai irányelve az egészségügyi technológia értékelés módszertanáról és ennek keretében költséghatékonysági
12. ZIN – Zorginstituut Nederland. Richtlijn voor het uitvoeren van economische evaluaties in de gezondheidszorg [Guideline for economic evaluations in healthcare]. Zorginstituut Nederland - National Health Care Institute; 2016 [updated June 2016. <https://www.zorginstituutnederland.nl/binaries/zinl/documenten/publicatie/2016/02/29/richtlijn-voor-het-uitvoeren-van-economische-evaluaties-in-de-gezondheidszorg/richtlijn-voor-het-uitvoeren-van-economische-evaluaties-in-de-gezondheidszorg.pdf>. Accessed 10 May 2020.
13. López-Bastida J, Oliva J, Antoñanzas F, García-Altés A, Gisbert R, Mar J, et al. Spanish recommendations on economic evaluation of health technologies. Eur J Health Econ. 2010;11(5):513-20.
14. López Bastida J, Oliva J, Antoñanzas F, García-Altés A, Gisbert R, Mar J, et al. Propuesta de guía para la evaluación económica aplicada a las tecnologías sanitarias 2010 [154-70]. <http://scielo.isciii.es/scielo.php?script=sci_arttext&pid=S0213-91112010000200012&nrm=iso>. Accessed 10 May 2020.
15. EUnetHTA. Methods for health economic evaluations - A guideline based on current practices in Europe: Swedish Council on Health Technology Assessment (SBU)/Sweden; 2015. <https://www.eunethta.eu/wp-content/uploads/2018/03/Methods_for_health_economic_evaluations.pdf>. Accessed 10 May 2020.
16. Mora-Ripoll R, Gilabert-Perramon A, Oliva-Moreno J, Puig-Junoy J. Guidance For Economic Evaluation and Budget Impact Analysis For Pharmaceuticals in Catalonia (Spain). Value Health. 2014;17(7):A447.
17. Jones K, Burns A. Costs of Health and Social Care 2021. 2021. <https://www.pssru.ac.uk/project-pages/unit-costs/unit-costs-of-health-and-social-care-2021/>. Accessed 21 Sept 2021.
18. NHS – National Health Service. National Cost Collection for the NHS 2021. <https://www.england.nhs.uk/costing-in-the-nhs/national-cost-collection/#ncc1819>. Accessed 10 May 2020.
19. NHS – National Health Service. Approved Costing Guidance: NHS Improvement; 2018 [updated 1 May 2018. <https://improvement.nhs.uk/resources/approved-costing-guidance/>. Accessed 10 May 2020.
20. Krauth C, Hessel F, Hansmeier T, Wasem J, Seitz R, Schweikert B. Empirische Bewertungssätze in der gesundheitsökonomischen Evaluation-ein Vorschlag der AG Methoden der gesundheitsökonomischen Evaluation (AG MEG) [Empirical standard costs for health economic evaluation in Germany -- a proposal by the working group methods in health economic evaluation]. Gesundheitswesen. 2005;67(10):736-46.
21. Braun S, Prenzler A, Mittendorf T, Schulenburg JMvd. Bewertung von Ressourcenverbräuchen im deutschen Gesundheitswesen aus Sicht der Gesetzlichen Krankenversicherung [Appraisal of Resource Use in the German Health-Care System from the Perspective of the Statutory Health Insurance]. Gesundheitswesen. 2009;71(01):19-23.
22. Prenzler A, Zeidler J, Braun S, von der Schulenburg J-M. Bewertung von Ressourcen im Gesundheitswesen aus der Perspektive der deutschen Sozialversicherung. Pharmacoeconomics German Research Articles. 2010;8(1):47-66.
23. Lange A, Prenzler A, von der Schulenburg J-MG. Die Perspektive der Privaten Krankenversicherung bei der Bewertung von Ressourcenverbräuchen im Rahmen von gesundheitsökonomischen Evaluationen: Sinnhaftigkeit und Umsetzung. Gesundheitsökonomie & Qualitätsmanagement. 2012;17(04):185-91.
24. Bock JO, Luppa M, Brettschneider C, Riedel-Heller S, Bickel H, Fuchs A, et al. Impact of depression on health care utilization and costs among multimorbid patients--from the MultiCare Cohort Study. PLoS One. 2014;9(3):e91973.
25. Bock JO, Brettschneider C, Seidl H, Bowles D, Holle R, Greiner W, et al. Ermittlung standardisierter Bewertungssätze aus gesellschaftlicher Perspektive für die gesundheitsökonomische Evaluation [Calculation of standardised unit costs from a societal perspective for health economic evaluation]. Gesundheitswesen. 2015;77(1):53-61.
26. Bock J-O, Bowles D, Brettschneider C, Greiner W, Holle R, König H-H, et al. Standardisierte Bewertungssätze aus gesellschaftlicher Perspektive für die gesundheitsökonomische Evaluation: Nomos Verlagsgesellschaft mbH & Co. KG; 2015.
27. Grupp H, König H-H, Konnopka A. Kostensätze zur monetären Bewertung von Versorgungsleistungen bei psychischen Erkrankungen. Gesundheitswesen. 2017;79(1):48-57.
28. Scholz S, Biermann-Stallwitz J, Brettschneider C, Damm O, Freytag A, Greiner W, et al. Standardisierte Kostenberechnungen im deutschen Gesundheitswesen: Bericht der Arbeitsgruppe „Standardkosten “des Ausschusses „ökonomische Evaluation “der dggö. Gesundheitsökonomie & Qualitätsmanagement. 2020;25(01):52-9.
29. Oostenbrink JB, Koopmanschap MA, Rutten FF. Standardisation of costs: the Dutch Manual for Costing in economic evaluations. Pharmacoeconomics. 2002;20(7):443-54.
30. Tan SS, Bouwmans CAM, Rutten FFH, Hakkaart-van Roijen L. Update of the Dutch manual for costing in economic evaluations. Int J Technol Assess Health Care. 2012;28(2):152-8.
31. Drost RMWA, Paulus ATG, Ruwaard D, Evers SMAA. Handleiding intersectorale kosten en baten van (preventieve) interventies: Classificatie, identificatie en kostprijzen. Maastricht, Netherlands: Maastricht University; 2014.
32. Bendeck M, Serrano-Blanco A, García-Alonso C, Bonet P, Jordà E, Sabes-Figuera R, et al. An integrative cross-design synthesis approach to estimate the cost of illness: An applied case to the cost of depression in Catalonia. J Ment Health. 2013;22(2):135-54.
33. Generalitat de Catalunya. Cost i càrrega de la depressióa Catalunya [Cost and burden of depression in Catalonia] Barcelona: Departament de Salut, Generalitat de Catalunya; 2010. <https://scientiasalut.gencat.cat/bitstream/handle/11351/2670/estudi_costos_carrega_depressio_catalunya_2011_ca.pdf?sequence=5>. Accessed 10 May 2020.
34. ISPOR. Pharmacoeconomic Guidelines Around the World - Austria 2018 [updated 16 Jan 2018]. <https://www.ispor.org/PEguidelines/countrydet.asp?c=29&t=4>. Accessed 10 May 2020.
35. ISPOR. Pharmacoeconomic Guidelines Around the World - England & Wales 2018 [updated 8 Feb 2018]. <https://www.ispor.org/PEguidelines/countrydet.asp?c=23&t=2>. Accessed 10 May 2020.
36. ISPOR. Pharmacoeconomic Guidelines Around the World - Germany 2013 [updated 1 Oct 2013]. <https://www.ispor.org/PEguidelines/countrydet.asp?c=9&t=1>. Accessed 10 May 2020.
37. ISPOR. Pharmacoeconomic Guidelines Around the World - Hungary 2018 [updated 12 Jan 2018]. <https://www.ispor.org/PEguidelines/countrydet.asp?c=10&t=4>. Accessed 10 May 2020.
38. ISPOR. Pharmacoeconomic Guidelines Around the World - Netherlands 2018 [updated 15 Jan 2018]. <https://www.ispor.org/PEguidelines/countrydet.asp?c=22&t=1>. Accessed 10 May 2020.
39. ISPOR. Pharmacoeconomic Guidelines Around the World - Spain 2018 [updated 12 Apr 2018]. <https://www.ispor.org/PEguidelines/countrydet.asp?c=20&t=4>. Accessed 10 May 2020.
40. Bouwmans C, De Jong K, Timman R, Zijlstra-Vlasveld M, Van der Feltz-Cornelis C, Tan Swan S, et al. Feasibility, reliability and validity of a questionnaire on healthcare consumption and productivity loss in patients with a psychiatric disorder (TiC-P). BMC Health Serv Res. 2013;13:217.
41. Bouwmans C, Krol M, Severens H, Koopmanschap M, Brouwer W, Roijen LH-v. The iMTA Productivity Cost Questionnaire: A Standardized Instrument for Measuring and Valuing Health-Related Productivity Losses. Value Health. 2015;18(6):753-8.
42. Hoefman RJ, van Exel J, Brouwer W. How to include informal care in economic evaluations. Pharmacoeconomics. 2013;31(12):1105-19.
